# Efficacy, safety, and cost-effectiveness analysis of adjuvant herbal medicine treatment, Palmijihwang-hwan, for chronic low back pain: a study protocol for randomized, controlled, assessor-blinded, multicenter clinical trial

**DOI:** 10.1186/s13063-019-3776-7

**Published:** 2019-12-27

**Authors:** Won-Suk Sung, Sae-Rom Jeon, Ye-Jin Hong, Tae-Hun Kim, Seungwon Shin, Hyun-Jong Lee, Byung-Kwan Seo, Yeon-Cheol Park, Eun-Jung Kim, Dong-Woo Nam

**Affiliations:** 1 Department of Acupuncture & Moxibustion, Dongguk University Bundang Oriental Hospital, Bundang-gu, Seongnam-si, Gyeonggi-do 13601 South Korea; 2 Department of Clinical Korean Medicine, Graduate School, Kyung Hee University, Seoul, South Korea; 3 Clinical Trial Center, Korean Medicine Hospital, Department of Korean Medicine, Kyung Hee University, Seoul, South Korea; 4 Department of Acupuncture & Moxibustion, College of Korean Medicine, Daegu Haany University, Gyeongsan-si, Gyeongsangbuk-do South Korea; 5 Department of Acupuncture & Moxibustion, College of Korean Medicine, Kyung Hee University, 26 Kyungheedae-ro, Dongdaemun-gu, Seoul 02447 South Korea

**Keywords:** Chronic low back pain, Palmijihwang-hwan, Randomized controlled trial, Study protocol

## Abstract

**Background:**

Low back pain is a common symptom and continuous or recurrent pain results in chronic low back pain (CLBP). While many patients with CLBP have tried various treatments, complementary and alternative medicine including acupuncture and herbal medicine is one of the commonly used treatments. Palmijihwang-hwan is a herbal medicine used frequently in clinical practice but there has been no report of the efficacy, safety, or cost-effectiveness analysis of Palmijihwang-hwan for CLBP.

**Methods:**

This study is a randomized, assessor-blinded, multicenter, clinical trial with two parallel groups. Four Korean medicine hospitals will recruit 84 participants and randomly allocate them into the control or treatment group in a 1:1 ratio. The control group will receive acupuncture treatment at 11 local and 4 distal acupuncture points for 20 min twice a week for 6 weeks. The treatment group will receive the same acupuncture treatment as the control group and also take Palmijihwang-hwan for 6 weeks. The primary outcome will be the change in visual analog scale (VAS) score between baseline (visit 1) and completion of the intervention (visit 12), and secondary outcomes will be pain-related clinical relevance (minimal clinical important difference or the proportion of the participants who decrease more than 30, or 50% on VAS), disability (Roland and Morris Disability Questionnaire), quality of life (EuroQol-5D), global assessment (Patient Global Impression of Change), and economic analysis (cost-effectiveness and cost-utility analysis). Additionally, safety will be assessed.

**Discussion:**

The results of our study will provide the clinical evidence about the efficacy, safety, and cost-effectiveness analysis of Palmijihwang-hwan for CLBP. There will be a chance to provide multiple subdivided influence of this treatment with various outcome measures, but lack of placebo is our limitation.

**Trial registration:**

Clinical Research Information Service, KCT0002998. Registered on 12 July 2018.

## Background

Low back pain (LBP) is a symptom that 84% of humans experience at some time in their life [1]. LBP can be classified according to its duration into acute (< 6 weeks), subacute (6–12 weeks), and chronic (> 12 weeks) [2]. Most patients with acute LBP recover within 4 weeks but recurrences are common [3]. In the case of insufficient treatment and management, continuous pain results in chronic LBP (CLBP), which is known to be related to various factors including psychological distress and psychiatric disorders [4]. Deficits in and poor satisfaction with conventional treatments [5] for CLBP have led to attempts to treat CLBP using nonpharmacologic [6] or complementary and alternative medicine (CAM).

In Korean medicine, several treatments including acupuncture [7], electroacupuncture [8], and pharmacopuncture such as bee venom [9] have been examined as a treatment for LBP, and several studies have reported therapeutic effects. Particularly, acupuncture is a well-known treatment for CLBP. Various studies have reported on the effects on pain [10] and the pragmatic aspects [11] of acupuncture in treating CLBP and some have suggested that acupuncture has more effect on the reduction of pain and bothersomeness than sham control in patients with CLBP [12]. Several studies have investigated the clinical effect of acupuncture combined with other treatments such as direct moxibustion [13], physiotherapy [14], and pharmacopuncture [15]. However, studies on the effects of adjuvant Korean herbal medicines on CLBP, particularly in combination with acupuncture, are rare.

Among them, Palmijihwang-hwan (Ba-Wei-Di-Huang-Wan in Chinese and Hachimi-jio-gan in Japanese) has been reported to be useful for skeletal muscle proliferation [16] and to be helpful for patients with sciatic neuralgia or lumbago with bad circulation in the lower body [17], which is similar to the symptoms of CLBP. Eight herbs used in Palmijihwang-hwan are also reported to have analgesic effects in experimental studies. It is reported that *Aconitum carmichaelii* improves inflammatory conditions by mediating TNF-alpha response [18] and processed *A. carmichaelii* exerts an analgesic effect via central opioid receptors [19]. Catapol, an ingredient in *Rehmannia glutinosa*, alleviates neuropathic pain with the modulation of neuroinflammation in the spinal cord in rats [20]. *Cornus officinalis* is reported to have anti-inflammatory and analgesic effects [21], while *Dioscorea batatas* is confirmed to have anti-inflammatory activity through the inhibition of iNOS and COX-2 expression [22]. Scientific studies indicate the anti-inflammatory effects of *Alisma orientale* [23]*, Poria coccos* [24]*, Paeonia sulffruticosa* [25]*, and Cinnamomum cassia* [26].

However, these reports include no clinical study to confirm the efficacy or safety of Palmijihwang-hwan or to include a cost-effectiveness analysis (CEA) of Palmijihwang-hwan, for treatment of CLBP. Therefore, we describe a protocol for a randomized controlled clinical trial that uses Palmijihwang-hwan combined with acupuncture for treatment of CLBP.

## Methods and design

### Objectives

The purpose of this study is to investigate the efficacy, safety, and CEA of Palmijihwang-hwan combined with acupuncture for treatment of CLBP by comparing this with the effects of acupuncture treatment alone.

### Design

**Fig. 1:**
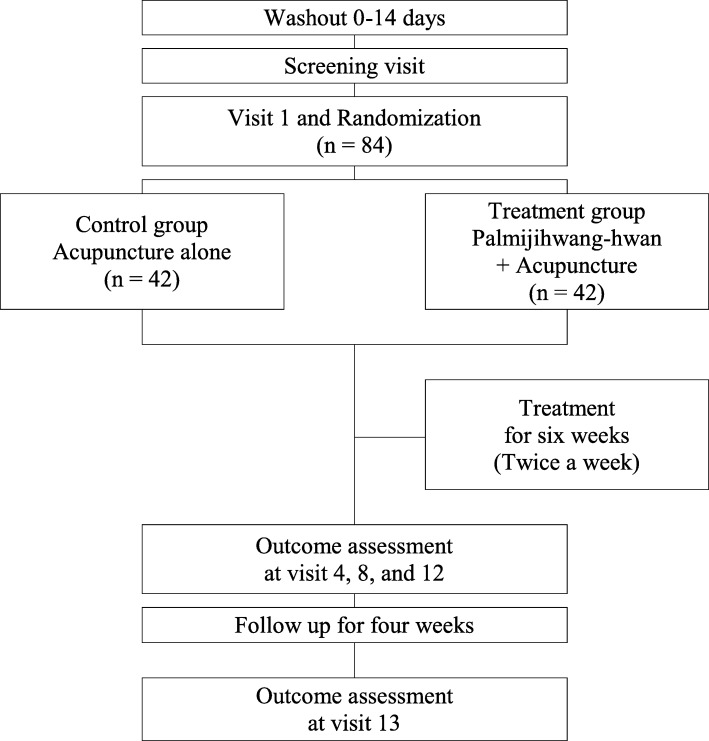
Study flowchart

This study is a randomized, assessor-blinded, multicenter, clinical trial with two parallel groups. We intend to investigate the synergic effects of Palmijihwang-hwan with acupuncture treatment in patients with CLBP. Eligible participants will be assigned to one of two groups, the control group (only acupuncture) or the treatment group (Palmijihwang-hwan + acupuncture). The flowchart is as shown in Fig. 1. The Standard Protocol Items: Recommendations for Interventional Trials (SPIRIT) checklist is provided in Additional file 1.

### Sample size calculation

We performed sample size calculations to determine the appropriate number of participants. The purpose of this study is to verify the synergic effect of the combination treatment (acupuncture + Palmijihwang-hwan) compared to acupuncture treatment alone, using a visual analog scale (VAS) score for change between baseline and completion of the intervention (6 weeks). However, no study has been conducted to investigate the effects of Palmijihwang-hwan combined with acupuncture in the treatment of CLBP. Thus, we reviewed a similar study conducted in patients with CLBP [27] in which the authors evaluated the efficacy of a herbal formula, Ojeok-san, by measuring the VAS score change between baseline and completion of the intervention (4 weeks) and comparing this with the placebo group in randomized clinical trials of CLBP. In that study, the change in VAS score between baseline and completion of the intervention was − 26.41 ± 19.06 in the treatment group and − 12.83 ± 19.45 in the control group. Based on these data, 33 participants would be required per intervention group for a two-arm study and a two-tailed test with 0.80 power (1-β) at the 0.05 α level, as calculated using G* Power (Version 3.1.9.3) [28]. After assuming a 20% drop out rate, we calculated that we would need to recruit 42 participants per group. Thus, a total of 84 participants will be required.

### Participants

We will recruit 84 participants with CLBP and assign them randomly to the control group or the treatment group in a 1:1 ratio.

#### Inclusion criteria

#### The inclusion criteria are:

1. Patients with CLBP, aged 19–65 years
2. Chief complaint of LBP lasting > 3 months
3. Score > 4 cm on a 10-cm VAS for LBP at one week prior to enrollment
4. Voluntarily agreeing to participation and observance of trials with written consent

#### Exclusion criteria

#### The exclusion criteria are:

1. Hypersensitive reactions to or history of reactions to Palmijihwang-hwan or its components
2. Abnormalities in the lower extremity on neurological examination
3. Requirement for surgical treatment because of severe neurological deficits (sensory or motor) or cauda equina syndrome
4. Previous spinal surgery or scheduled spinal surgery during the trial
5. Patients with scoliosis or neurodegenerative disease
6. Vertebral fracture, inflammatory spondylitis, spinal infection, or malignant tumor
7. Pregnancy, lactation, plans to conceive, refusal to use appropriate contraception methods during the trial
8. Gastrointestinal disorders or diarrhea
9. Genetic disorders such as galactose intolerance, Lapp lactase deficiency, or glucose-galactose malabsorption
10. Use of acupuncture, herbal medicine, injection, physical therapy, or manipulation treatment for low back pain within two weeks
11. Chief complaint of part other than the lumbar region
12. Participants in other clinical studies related to CLBP within a month
13. Ineligibility for the trial as judged by the investigator

### Recruitment

Four Korean medicine hospitals, Kyung Hee University Korean Medicine Hospital, Kyung Hee University Korean Medicine Hospital at Gangdong, Oriental Medicine Hospital of Daegu Haany University, and Dongguk University Bundang Oriental Hospital, will recruit participants through advertisements on bulletin boards, local newspapers, and public boards.

### Procedure

**Fig. 2:**
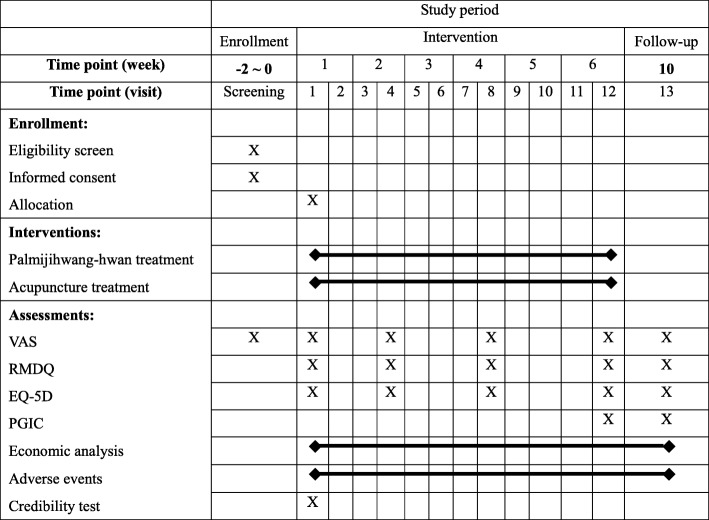
Study schedule (Standard Protocol Items: Recommendations for Interventional Trials, SPIRIT). Abbreviations: EQ-5D: EuroQol-5 dimension; PGIC: patient global impression of change; RMDQ: Roland and Morris disability questionnaire; VAS: visual analog scale

The study schedule is as shown in Fig. 2. At the screening visit, patients who are interested in participation and who visit one of the four Korean medicine hospitals will be informed about the study. They will be also informed about expected benefits and risks and that they are able to withdraw from the study at any visit. If they sign the informed consent form voluntarily, the investigator will perform several examinations including check of vital signs, physical examination, spine x-ray, blood chemistry test, pregnancy test, and will collect demographic data, CLBP history, and concomitant treatment data, and apply the VAS, to determine the patient’s eligibility. If patients are eligible for the study but are taking medication, the investigator will determine the day of screening according to the participant’s medication to give a washout period. If the participant is taking anti-inflammatory medication or painkillers, the investigator will give a 2-week washout period. During the 6-week treatment, the participants in both groups will visit the hospital twice a week (total 12 visits) and receive acupuncture treatment at every visit. Participants in the treatment group will additionally receive Palmijihwang-hwan (at visit 1, 4, and 8) during the treatment period. Then, participants in both groups will be asked to complete a survey every 2 weeks (at visit 4, 8, and 12). On visit 13, 4 weeks after visit 12, all participants will visit the hospital for the final time.

### Randomization and allocation concealment

A randomization sequence will be generated by an independent statistician who does not participate in this study, using R software (Version 3.5.0; R Foundation for Statistical Computing, Vienna, Austria). Randomization will be stratified based on four Korean medicine hospitals, and then block randomization will be used to approximately equalize the number of participants in each group. A random code will be delivered in the sealed envelopes, in which the allocated group is written. A delegated investigator in each site will open the envelope in sequence and allocate a participant to either intervention or control arm, only when all the screening criteria are satisfied. Allocation will be concealed until the investigator opens each sealed envelope. The randomization sequence will be kept by the independent statistician or physician during the clinical trial period and concealed until the occurrence of the event predefined as a reason for code-breaking.

### Blinding

The additional Palmijihwang-hwan treatment means that the participants and practitioners cannot be blinded; thus, the practitioners who perform the acupuncture treatment or provide Palmijihwang-hwan will not assess the outcome. The assessor who record case report form (CRF) will be instructed to ask questions simply and write in detail the participant's answer. Both of the assessor and participant are prevented from talking about the treatment.

### Interventions

#### Control group intervention

Participants in the control group will receive only the acupuncture treatment. Sterilized stainless steel needles with 0.25 mm width and 40 mm length (DB108C; Dongbang Medical Co., Boryung-si, South Korea) will be inserted at 11 local and 4 distal acupuncture points for 20 min. Acupuncture points are as shown in Table 1. Participants will receive the acupuncture treatment twice a week for 6 weeks, performed by Korean medical doctors who are specialists in acupuncture and moxibustion.

**Table 1:** Acupuncture treatment protocol based on the Standards for Reporting Interventions in Clinical Trials of Acupuncture (STRICTA) [29]

1. Acupuncture rationale	1a) Acupuncture technique	Traditional Korean medicine theory
	1b) Reasoning for treatment provided (based on historical context, literature sources, and/or consensus methods), with where appropriate	Textbook of acupuncture and moxibustion and related articles
	1c) Extent of treatment variations	EX-B2 of lesion or upper and lower levels
2. Details of needling	2a) Number of needle insertions per subject per session	Acupuncture points: 15
	2b) Names of points used	Local acupuncture points: GV3, EX-B5/bilateral BL23, BL24, BL25, BL26 (total 11 points) Distal acupuncture points: bilateral BL40, BL60 (total 4 points)
	2c) Depth of insertion based on a specified unit of measurement	20–40 mm
	2d) Response sought	De-qi (penetrating, sharp, aching and painful sensations)
	2e) Needle stimulation	Rotation of the acupuncture needle 3–5 times
	2f) Needle retention time	20 min
	2 g) Needle type	Sterilized stainless steel needle with 0.25 mm width and 40 mm length (DB108C; Dongbang Medical Co., Boryung-si, South Korea)
3. Treatment regimen	3a) Number of treatment sessions	12 sessions
	3b) Frequency and duration of treatment sessions	Twice a week for six weeks (Total 12 times)
4. Other components of treatment	4a) Details of other interventions used for the treatment group	Lifestyle advice about chronic low back pain
	4b) Setting and context of treatment, including instructions to practitioners and information and explanations for patients	Practitioners are instructed to prevent talking with participants
5. Practitioner background	5) Description of participating acupuncturists	Korean medical doctors who are specialist in acupuncture and moxibustion at least three years of experience, or under supervision by a specialist
6. Control interventions	6a) Rationale for the control or comparator in the context of the research question with sources that justify this choice	Textbook of acupuncture and moxibustion and related articles
	6b) Precise description of the control or comparator	Same acupuncture treatment in two groups: not applicable
	If sham acupuncture or any other type of acupuncture-like control is used, provide details as for items 1–3.	Same acupuncture treatment in two groups

#### Treatment group intervention

Participants in the treatment group will receive the same acupuncture treatment as the control group and will also take Palmijihwang-hwan. At visit 1, 4, and 8, they will receive 2-week doses of Palmijihwang-hwan (14 days + an extra 7 days) in an individual package from the practitioners. The Palmijihwang-hwan that will be used in this study is a brown, bitter herbal extract granule produced and packed by Kracie Pharma Korea Co., Ltd. (Seoul, Korea) in accordance with the Korean Good Manufacturing Practice guidelines. Palmijihwang-hwan contains eight herbs (listed in Table 2), is water-extracted with starch and lactose, and is regulated by the Korean Food & Drug Administration. At the production stage, each herb is tested for properties, morphology, and undergoes physicochemical and microorganism examination. Then Palmijihwang-hwan is produced by extraction, separation, concentration, drying, and granulation with compound herbs. Multiple quality tests are performed, including pattern analysis of the ingredients contained to ensure quality and stability, and we will test for residual pesticides to ensure product safety. Palmijihwang-hwan, packed for one dose, will be provided by an independent pharmacist in a separate room and participants will be instructed to take it with water 30 min after breakfast and after dinner, for 6 weeks.

**Table 2:** Ingredients of Palmijihwang-hwan

Scientific name	Proportion
*Rehmannia glutinosa (steamed 9 times)*	5.0
*Cornus officinalis*	3.0
*Dioscorea batatas*	3.0
*Alisma orientale*	3.0
*Poria coccos*	3.0
*Paeonia sulffruticosa*	3.0
*Cinnamomum cassia*	1.0
*Aconitum carmichaelii (processed)*	1.0

#### Concomitant treatment

Other interventions including muscle relaxants, non-steroidal anti-inflammatory drugs (NSAIDs) (oral medication, topical application, or patch), antidepressant agents, anticonvulsants, treatments with Korean medicine (acupuncture, herbal medicine, and cupping), physical therapy, injections, or surgery will not be permitted during the treatment period (but are permitted during the follow-up period). However, concomitant medications taken within 4 weeks before trial participation or that are considered to have no effect on the interpretation of the results of this study may be allowed dependent on the judgment of the investigator.

#### Adverse events

Adverse events will be checked at every visit. If adverse events occur, the practitioners will record them in detail in the CRF and decide upon trial continuation depending on the symptoms. Abnormal change in blood chemistry tests, vital signs, or change of medication/treatment will also be checked.

### Outcome measurement

#### Primary outcome

The primary outcome of this study will be change in the VAS score between baseline (visit 1) and completion of the intervention (visit 12) [30]. Using the 10-cm VAS (0, absence of pain; 10, the worst pain imaginable), the participants will be asked to report their degree of LBP.

#### Secondary outcomes

#### The secondary outcomes are:

1. Pain: change in the VAS score between baseline (visit 1) and week 2 (visit 4), week 4 (visit 8), week 6 (visit 12), and 4 weeks after completion of the intervention (visit 13). Additional clinical relevance will be compared as follows;
  - Based on previous studies [31, 32], the proportion of the minimal clinically important difference (MCID) in CLBP is defined as a 2 cm decrease on the VAS. The proportion of MCID (more than 2 cm decrease on the VAS) between baseline and visit 4, 8, 12, and 13 will be recorded.
  - The proportion of participants who experience more than 30% decrease on the VAS between baseline and visit 4, 8, 12, and 13.
  - The proportion of participants who experience more than 50% decrease on the VAS between the baseline and visit 4, 8, 12, and 13.
2. Disability: to evaluate the physical disability due to low back pain, the Roland and Morris Disability Questionnaire (RMDQ) will be applied [33]. The RMDQ score is calculated by 24 items (in the range 0 (no disability) to 24 (maximum disability)). Change in score in the Korean version of the RMDQ will be compared between baseline and visit 4, 8, 12, and 13.
3. Quality of life: to evaluate quality of life, the EuroQol-5D (EQ-5D) questionnaire will be applied. The EQ-5D measures five dimensions (mobility, self-care, activities of daily life, pain, and anxiety/depression) using a 1–3 scale, and a VAS to rate the current health state using a 0–100 scale [34]; change in the EQ-5D scores between baseline and visit 4, 8, 12, and 13 will be compared.
4. Global assessment: to evaluate participants’ impressions of change, the Patient Global Impression of Change (PGIC) will be applied at visit 12 and 13. The PGIC is measured on 7-point scale (from 1 (completely recovered) to 7 (vastly worsened)) with a score of 1–2 indicating improvement, 3–5 no change, and 6–7 deterioration [35].
5. Economic analysis: to evaluate the intervention-related cost, CEA and cost-utility analysis (CUA) will be conducted. Cost data will be collected separately as follows;
  - Medical cost: official medical costs will include benefit coverage by National health insurance and out-of-pocket payments such as for drugs and therapeutic equipment when visiting clinics or hospitals, for treating CLBP. Unofficial medical costs will include payments for over-the-counter drugs, dietary supplements, medical devices, orthotic devices, etc. which may be used without prescriptions by physicians.
  - Non-medical cost: transportation costs, care costs, time costs, and lost productivity costs.
6. Credibility test: to evaluate the credibility of Palmijihwang-hwan, a credibility/expectancy questionnaire will be applied at visit 1. Using a 9-point scale, a higher score indicates higher credibility and expectancy of Palmijihwang-hwan [36]

### Safety assessment

Physical examinations will be performed at the screening visit and at visit 1 and 13. Blood chemistry tests including pregnancy tests will be conducted at the screening visit and visit 12. The blood chemistry test will assess the red blood cell (RBC) count, white blood cell (WBC) count, hemoglobin (Hb), hematocrit (Hct), platelet count, erythrocyte sedimentation rate (ESR), aspartate aminotransferase (AST), alanine aminotransferase (ALT), blood urea nitrogen (BUN), creatinine, electrolytes (Na, K, Cl), and C-reactive protein (CRP). Vital signs, adverse events, and change in medication/treatment will be checked at every visit.

### Withdrawal criteria

### The withdrawal criteria are:

1. Violation of the inclusion criteria or applicable to exclusion criteria
2. Serious adverse events that make it difficult to maintain the trial
3. Refusal to continue, withdrawal of consent by the participants and/or legal representative
4. Compliance with Palmijihwang-hwan or acupuncture treatment less than 80% (participants should take more than 23 of the total 28 doses of Palmijihwang-hwan given at the time of the visit and receive more than 10 of the total 12 acupuncture treatments)
5. Violation of the clinical trial protocol by the investigator or the participants
6. Loss to follow up
7. Use of medications or treatments that affect the results of the trials without the permission from the practitioner
8. Inappropriate process as judged by the investigator

### Data management and quality control

To guarantee the consistency of the clinical trial among the institutions, all staff participating in this trial will receive training before study initiation. This training will include education about the procedures, acupuncture treatment, and outcome measure assessments.

A clinical research coordinator, trained in good clinical practice, will collect and record the study data in the CRF. To ensure confidence in the data, study-related documents including consent forms, the CRF, questionnaires, medical records, and other records will be stored in a locked space or on a password-protected computer in each hospital for 3 years after study completion.

If the protocol is revised, this will be handled by Kyung Hee University Korean Medicine Hospital as a central coordinating facility. Any protocol amendments will be reviewed and approved by the ethics committee before application in the study and published via the Clinical Research Information System (CRIS).

No formal data monitoring committee will be convened for this study. However, quality will be maintained in the study by an independent clinical research associate (CRA) who will regularly audit and monitor the study at each hospital. The CRA will monitor the study on site to protect participants’ rights, assess compliance with the protocol, and ensure the ongoing implementation of appropriate data entry and quality control procedures.

### Statistical analysis

Continuous variables at baseline will be expressed as means plus/minus standard deviation and statistically tested using the two-sample *t* test or the Wilcoxon rank sum test. Categorical variables at baseline will be expressed as frequencies and statistically tested using the chi-squared test or Fisher’s exact test. If there is a significant difference between groups, the baseline variable will be considered as a covariate for efficacy analysis.

The full analysis set (FAS) will be analyzed primarily, including participants who take at least one dose of Palmijihwang-hwan and complete at least one pain assessment. Also, the per-protocol set (PPS) will be subordinately analyzed, including participants who take more than 80% of the total Palmijihwang-hwan dose and complete the trial. In the FAS analysis, the missing data will be imputed using the method of last observation carried forward. The primary outcome (change in the VAS score between baseline and endpoint) will be tested by analysis of covariance (ANCOVA) or rank ANCOVA (according to normality or homoscedasticity) with factors of group and study site and covariates of baseline values. Continuous variables for the secondary outcomes will also be tested, respectively, by ANCOVA or rank ANCOVA. Categorical variables for the secondary outcomes will be tested by chi-squared test or Fisher’s exact test according to the number of the cells with a value of 5 or less in the contingency table. Every variable predefined in the outcome sections will be compared at each time point, respectively.

The safety set will include participants who receive at least one dose of Palmijihwang-hwan. In safety analysis, the frequency and ratio of adverse events will be applied and compared between the treatment group and the control group using the chi-squared test or Fisher’s exact test. Any abnormal change in blood chemistry tests from visit 1 to 12 will be expressed as frequency and percentage and compared between the treatment group and the control group using McNemar’s chi-squared test, and any change in vital signs between visit 1 and 12 and between visit 1 and 13 will be expressed as change scores and compared between the treatment group and the control group using the two-sample *t* test or the Wilcoxon rank-sum test. Statistical tests will be carried out at 5% significance level (two-sided), using R software (Version 3.5.0 or later; R Foundation for Statistical Computing, Vienna, Austria).

For CEA, combination treatment with Palmijihwang-hwan with acupuncture will be compared with acupuncture alone from a healthcare system perspective. The treatment cycle will be assumed to be 10 weeks. The treatment period (i.e. analysis period) can be the average life span if we build a model for the data analysis. If not, it is impossible to extrapolate the observations to the patient’s life span. Therefore, this part is omitted as of now. For health-related outcomes, the RMDQ will be used for CEA and the EQ-5D for CUA. The absolute cost-effectiveness ratio will be estimated for the combination treatment of Palmijihwang-hwan with acupuncture and for acupuncture monotherapy. The cost-effectiveness of the combination therapy will be evaluated based on the incremental cost-effectiveness ratio. All economic analysis will be performed using TreeAgePro R 1.1 (Williamstown, MA, USA, 2018).

### Ethics approval and registration

This study was approved by the Institutional Review Board of four hospitals (Kyung Hee University Korean Medicine Hospital; KOMCIRB-180413-HR-012, Kyung Hee University Korean Medicine Hospital at Gangdong; KHNMCOH 2018–04-001; Oriental Medicine Hospital of Daegu Haany University; DHUMC-D-18010-PRO-02, and Dongguk University Bundang Oriental Hospital; DUBOH 2018–0006), and registered in the Clinical Research Information Service (CRIS, KCT0002998) on 12 July 2018.

## Discussion

Despite the development of medicine, the prevalence of CLBP and related disabilities have increased. Conventional treatments such as medications have been reported to have both benefits and harms [37], and several CAM therapies have been used widely and have proven efficacy and safety [38].

In herbal medicine, a CAM therapy, several randomized controlled trials have been conducted and Gagnier et al [39] have reviewed the effects of several herbal medicines on CLBP. However, these studies investigated the use of single herbal medicines such as *Capsicum frutescens* (cayenne) and *Harpagophytum procumbens* (devil’s claw), and there has been no study of the complex herbal medicines that are mainly used in Korean medicine.

Palmijihwang-hwan was used in this study; this is a representative herbal formula for supplementing kidney deficiency in Korean medicine. Palmijihwang-hwan has been suggested to regulate immunity, peroxidation, and the aging process [40], and to have an effect on diverse diseases including diabetes mellitus [41], dementia [42], and chemotherapy-induced peripheral neuropathy [43].

In LBP, we suggested that Palmijihwang-hwan could help patients who have fatigue, cold sensation, lumbago, reduced muscle strength [44], constant pain, or discomfort behavior [45], which might be helpful in CLBP. In addition, kidney deficiency is one of the most commonly diagnosed causes of CLBP [46, 47]. In the absence of a study on the use of herbal medicine in CLBP, this study will be the opportunity to provide information on the usefulness of Palmijihwang-hwan. Pharmacologically, it may provide the clinical information about the toxicity of A. carmichaelii by safety assessment. The toxicity of A. carmichaelii was known that lowest oral dose was 0.2 g/kg and processed aconitum carmichaelii was 60 g/kg [48] while processed *A. carmichaelii* was 60 g/kg [49].

Furthermore, the features of this study such as the multicenter design and various outcome measures, including clinical relevance, quality of life, disability level, global assessment, and economic analysis, will improve the quality of the trial data and prevent bias. However, this study has several limitations. First, participants in the control group cannot be blinded because of the absence of a placebo, which could influence the study results. Second, we quoted Lee’s study for sample calculation but there is a difference in treatment duration. Participants in Lee's study [27] took Ojeok-san for 4 weeks, but this study needs participants to take Palmijihwang-hwan for 6 weeks. Third, there is no rescue therapy including drugs in the case of severe LBP, which could inhibit participants’ compliance. However, the results of this study will provide clinical evidence about the efficacy, safety, and CEA of Palmijihwang-hwan for treatment of CLBP, and this evidence will be useful for patients, physicians, researchers, and stakeholders.

## Trial status

The final protocol version is 2.1 and is dated 23 July 2018. Recruitment began on 17 August 2018 and is ongoing.

## Supplementary information

**Additional file 1.** SPIRIT 2013 Checklist: Recommended items to address in a clinical trial protocol and related documents.

## Data Availability

Data and material from this trial are available upon reasonable request and approval by the corresponding author.

## Abbreviations

ANCOVA: Analysis of covariance
CAM: Complementary and alternative medicine
CEA: Cost-effectiveness analysis
CLBP: Chronic low back pain
CRA: Clinical research associate
CRF: Case report form
CUA: Cost-utility analysis
EQ-5D: EuroQol-5 dimension
FAS: Full analysis set
LBP: Low back pain
MCID: Minimal clinically important difference
NSAID: Non-steroidal anti-inflammatory drug
PGIC: Patient global impression of change
PPS: Per-protocol set
RMDQ: Roland and Morris disability questionnaire
STRICTA: Standards for reporting interventions in clinical trials of acupuncture
TNF: Tumor necrosis factor
VAS: Visual analog scale
